# A human T-lymphotropic virus-1 carrier who developed progressive multifocal leukoencephalopathy following immunotherapy for sarcoidosis: a case report

**DOI:** 10.1186/s12883-023-03094-w

**Published:** 2023-02-02

**Authors:** Takashi Nagahori, Wataru Shiraishi, Masafumi Nishikawa, Ayano Matsuyoshi, Takenori Ogura, Yui Yamada, Kenta Takahashi, Tadaki Suzuki, Kazuo Nakamichi, Tetsuya Hashimoto, Taketo Hatano

**Affiliations:** 1 Department of Neurosurgery, Kokura Memorial Hospital, Fukuoka, Japan; 2 Department of Neurology, Kokura Memorial Hospital, Fukuoka, Japan; 3 Shiraishi Internal Medicine Clinic, Fukuoka, Japan; 4 Department of Pathology, Kokura Memorial Hospital, Fukuoka, Japan; 5 Department of Pathology, National Institute of Infectious Diseases, Tokyo, Japan; 6 Department of Virology, National Institute of Infectious Diseases, Tokyo, Japan

**Keywords:** Demyelination, Human T-lymphotropic virus-1 (HTLV-1), Progressive multifocal leukoencephalopathy (PML), Sarcoidosis, Case report

## Abstract

**Background:**

Progressive multifocal leukoencephalopathy (PML) is a devastating demyelinating disorder of the central nervous system caused by opportunistic infection of the JC virus (JCV).

**Case presentation:**

A 58-year-old Japanese woman was admitted to our hospital for aphasia. She had a 5-year history of untreated sarcoidosis and was a human T cell lymphotropic virus-1 (HTLV-1) carrier. Serum angiotensin-converting enzyme, soluble interleukin-2 receptor, lysozyme, and calcium levels were elevated. JCV-DNA was not detected in cerebrospinal fluid by PCR testing. Skin biopsy revealed noncaseating granuloma formation. Bilateral multiple nodular lesions were present on chest X-ray. Brain magnetic resonance imaging showed left frontal and temporal lesions without gadolinium enhancement. As we suspected that systemic sarcoidosis had developed into neurosarcoidosis, we started steroid and infliximab administration. After treatment, the chest X-ray and serum abnormalities ameliorated, but the neurological deficits remained. At 1 month after immunotherapy, she developed right hemiparesis. Cerebrospinal fluid was positive for prototype (PML-type) JCV on repeated PCR testing. Brain biopsy revealed demyelinating lesions with macrophage infiltration, atypical astrocytes, and JCV antigen-positive cells. We diagnosed her with PML and started mefloquine, leading to partial remission.

**Conclusions:**

Sarcoidosis and HTLV-1 infection both affect T cell function, especially CD4^+^ T cells, and may developped the patient’s PML. The comorbidity of sarcoidosis, PML, and HTLV-1 infection has not been reported, and this is the world’s first report of PML associated with HTLV-1 infection and sarcoidosis.

## Background

Progressive multifocal leukoencephalopathy (PML) is a demyelinating disease of the central nervous system caused by opportunistic infection with the JC virus (JCV). PML is induced by a dysfunction of cellular immunity and can be triggered by various conditions, such as a human immunodeficiency virus (HIV) infection, hematologic malignancy, and disease-modifying drugs for multiple sclerosis [1]. Here, we describe a patient with a history of untreated sarcoidosis, who was a human T lymphotropic virus-1 (HTLV-1) carrier, and who eventually developed PML. Following treatment for sarcoidosis, the patient’s neurological symptoms worsened. Thereafter, she was diagnosed with PML based on the findings of cerebrospinal fluid (CSF) examination and brain biopsy. T cell dysfunction caused by HTLV-1 and sarcoidosis was considered to affect the pathogenesis of PML. This is the world’s first case report of PML associated with HTLV-1 infection and sarcoidosis.

## Case presentation

**Fig. 1:**
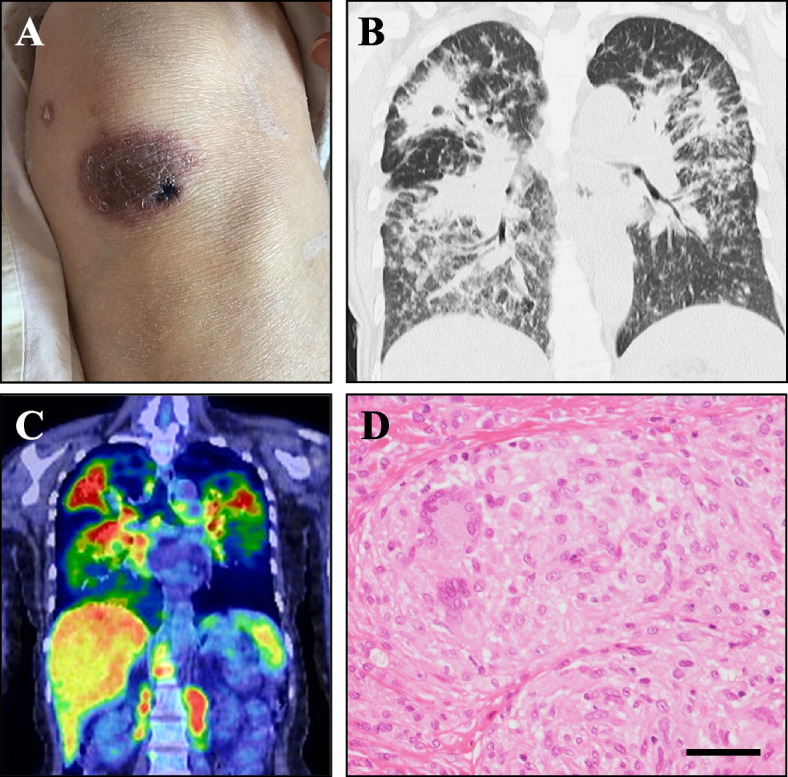
Skin lesions and chest imaging findings on admission. **A** A brownish skin lesion with a diameter of 3 cm was observed on the left knee. **B** Chest computed tomography revealed bilateral infiltrating band-like opaque lesions. **C** Chest positron emission tomography revealed diffuse accumulation in the lung lesions. **D** Skin biopsy hematoxylin–eosin (HE) staining showed noncaseating granuloma formation with multinucleated giant cells. Scale bar: 50 μm

**Fig. 2:**
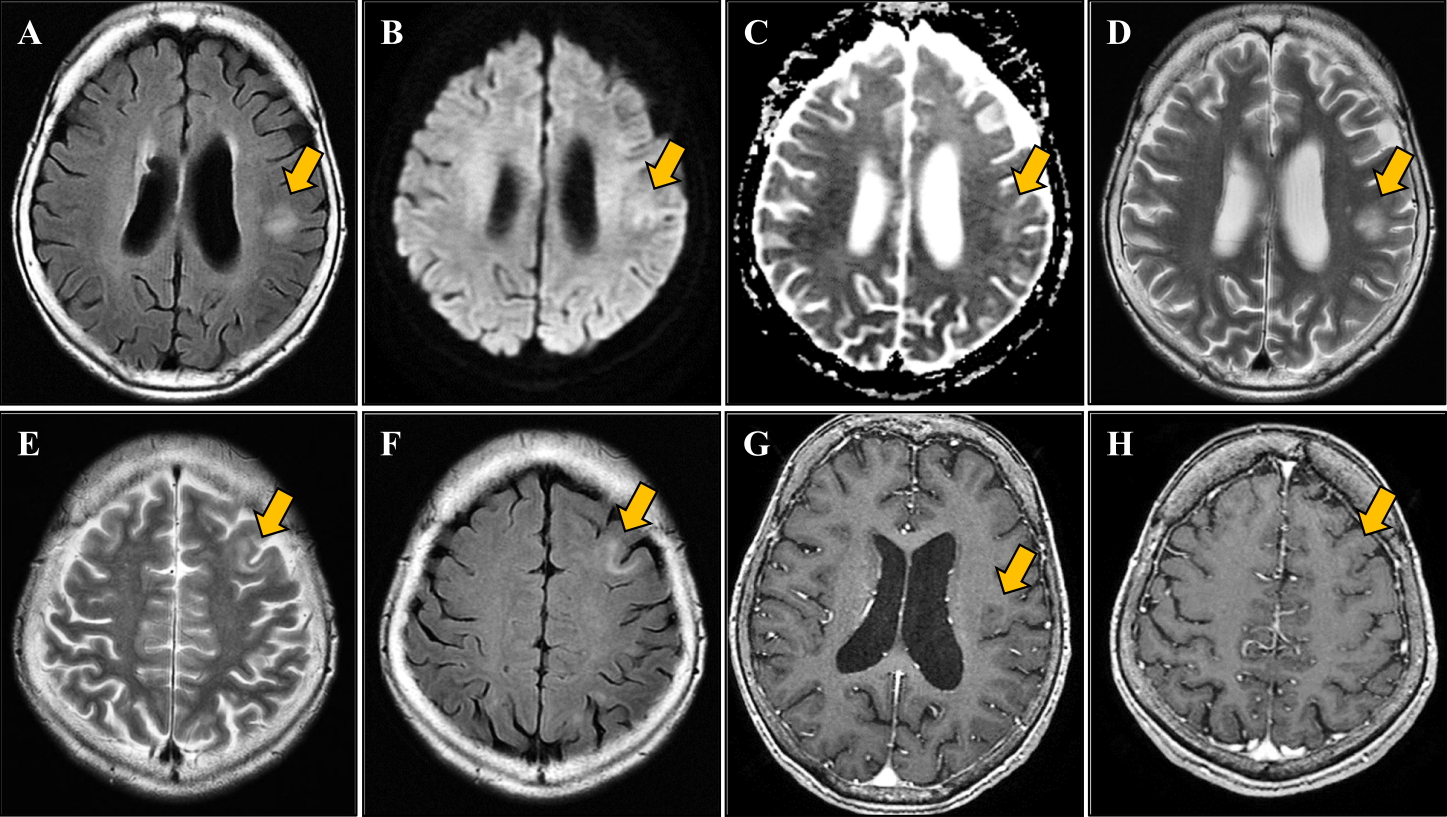
Brain magnetic resonance imaging on admission. Brain magnetic resonance imaging showed a hyperintense lesion in fluid-attenuated inversion recovery imaging (**A**), diffusion-weighted imaging (**B**), and apparent diffusion coefficient mapping (**C**) (arrows) in the left temporal lobe. T2-weighted images also show high-intensity lesions (**D**, **E**). There was also a left frontal lobe lesion (**F**, arrow). These lesions lacked gadolinium enhancement (**G**, **H**, arrows)

A 58-year-old Japanese female with a 5-year history of untreated sarcoidosis was admitted to our hospital due to progressive aphasia lasting for 4 months. She was an HTLV-1 carrier, but was not affected by leukemia. She was a current smoker with a Brinkman index of 560. Physical examination revealed fine crackles on the lower back and a brownish skin lesion on the left knee (Fig. 1A). On neurological examination, her Glasgow coma scale was 13/15 (E4V3M6). She showed mixed aphasia with a predominance of motor aphasia. There was no motor impairment on admission. Tendon reflexes showed hyperreflexia on the right side. Blood analysis revealed a normal white blood cell count with lymphocytopenia (770 cells/μL; normal: > 1,000 cells/μL) and elevation of angiotensin-converting enzyme (39.6 IU/L; normal: 7.7–29.4 IU/L), lysozyme (51.0 μg/mL; normal: 4.2–11.5 μg/mL), serum calcium (13.5 mg/dL; normal: 9.0–11.0 mg/dL), and soluble interleukin-2 receptor (3,080 U/mL; normal: 121–613 U/mL) levels. She was negative for serum HIV antigen/antibody, but she was positive for anti-HTLV-1 antibody. An interferon-gamma release assay for tuberculosis was negative. CSF analysis revealed no pleocytosis or protein elevation. CSF oligoclonal bands were negative, and cytopathology revealed no atypical or malignant cells. CSF was negative for JCV-DNA as judged by a highly sensitive real-time PCR test with a lower detection limit of 20 copies/mL. Chest X-ray showed bilateral diffuse nodular lesions, indicating sarcoidosis of chest X-ray stage IV [2]. Chest computed tomography and positron emission tomography (PET) revealed bilateral infiltrating lesions with PET marker accumulation (Fig. 1B, C). Ultrasound echocardiography and Holter monitoring revealed no signs of cardiac sarcoidosis. Skin biopsy of the knee lesion demonstrated noncaseating granuloma (Fig. 1D), a typical finding of sarcoidosis. Brain magnetic resonance imaging (MRI) indicated lesions in the left frontal and temporal lobes (Fig. 2A–H). These lesions were hyperintense in fluid-attenuated inversion recovery imaging, diffusion-weighted imaging, and apparent diffusion coefficient mapping without gadolinium enhancement.

**Fig. 3:**
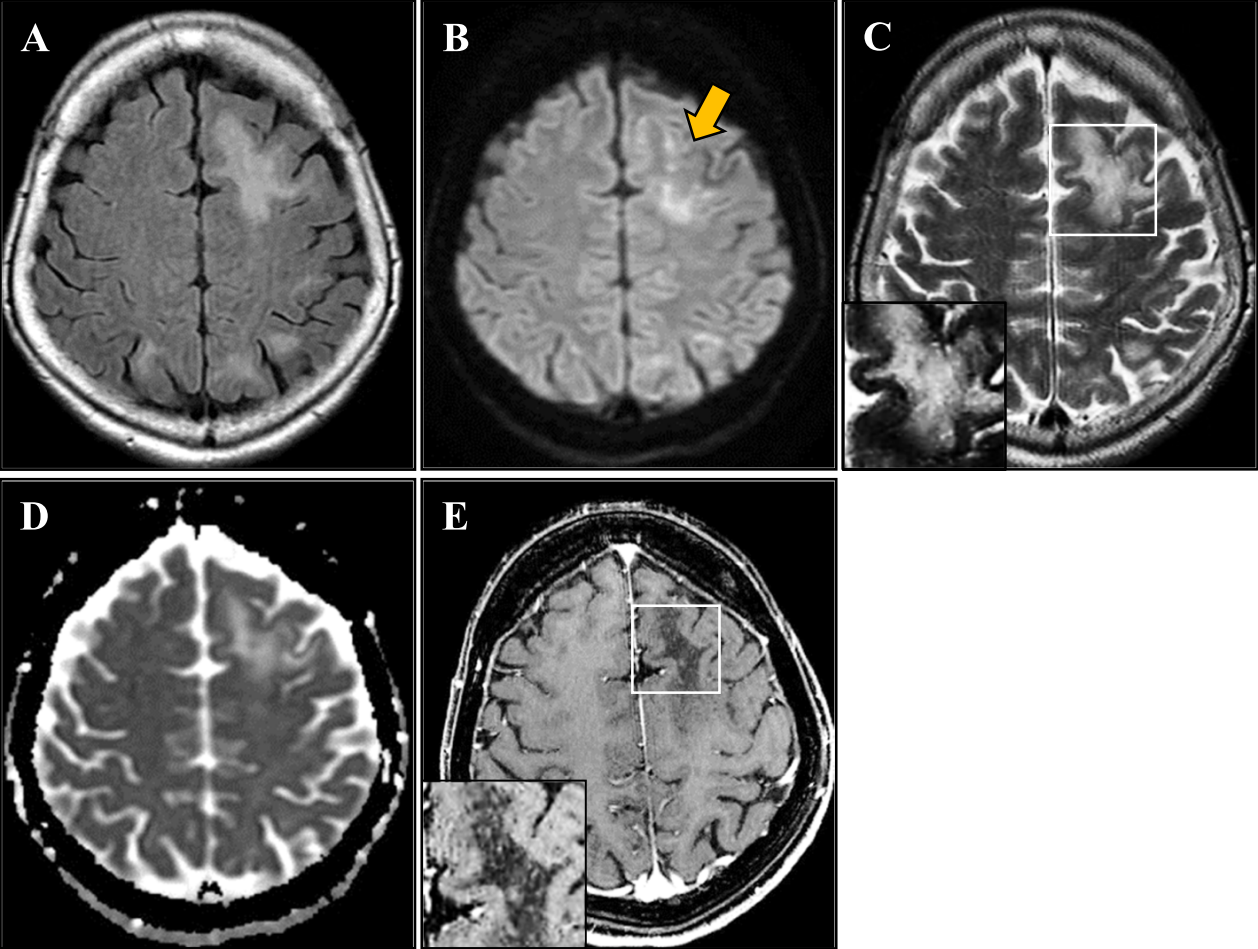
Brain magnetic resonance imaging after neurological deterioration. Brain magnetic resonance imaging showed an enlargement of the left frontal lobe lesion with hyperintensity in fluid-attenuated inversion recovery imaging (**A**), diffusion-weighted imaging (**B**), T2-weighted image (**C**, inset), and apparent diffusion coefficient mapping (**D**) with a “milky way appearance” (arrow). This lesion still lacked gadolinium enhancement (**E**) with “milky way appearance” in post-gadolinium enhancement (**E**, inset)

**Fig. 4:**
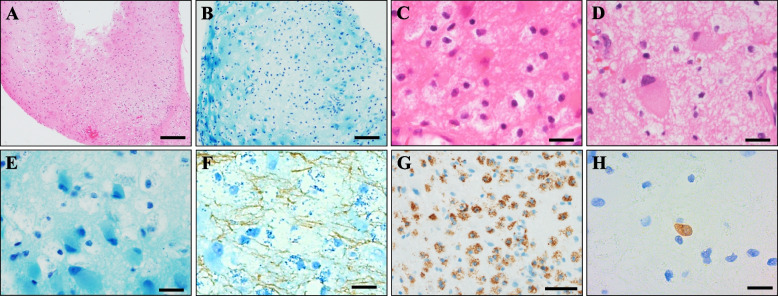
Pathological findings of a brain biopsy from the left frontal lesion. **A**, **B** In low-magnification view of hematoxylin–eosin (HE) and Klüver-Barrera (KB)-stained sections. The sampled lesion consisted of inflammatory cell infiltration and myelin loss. **C**, **D** High-magnification view of HE. HE staining showed foamy macrophage infiltration and atypical astrocytes. **E** KB staining demonstrated demyelination with myelin-laden macrophages. **F** Double-staining of KB and neurofilament immunostaining revealed relatively preserved axonal structures compared to myelin. **G** Immunostaining showed that the infiltrating cells were CD68 ^+^ . **H** Immunohistochemistry using an anti-JCV VP2/3 antibody indicated the presence of JC virus antigen-positive cells. Scale bars: 200 μm (**A**, **B**), 20 μm (**C**–**F**, **H**), and 50 μm (**G**)

On the basis of these results, we suspected the aphasia was caused by neurosarcoidosis in a patient with untreated sarcoidosis, and we initiated immunotherapy. Although she was negative for CSF JCV-DNA, we also started mirtazapine (15 mg/day), considering the possibility of PML based on the MRI findings. We administered steroid pulse therapy (methylprednisolone 1,000 mg/day × 3 days), followed by oral prednisolone (45 mg/day) with taper. After steroid treatment, infliximab (200 mg) was administered for the neurological symptoms. After initiating immunotherapy, her serum and chest X-ray findings were ameliorated, with the normalization of angiotensin-converting enzyme (9.8 IU/L), lysozyme (14.6 μg/mL), serum calcium (8.8 mg/dL), and soluble interleukin-2 receptor (356 U/mL) levels. However, her neurological conditions showed no improvement. She was transferred to a rehabilitation hospital with the continuation of 15 mg/day oral prednisolone. At 1 month later, right-sided total paresis appeared and she was re-admitted to our hospital. Brain MRI revealed scattered multifocal lesions in the left frontal lobe with a “milky way appearance” [3] (Fig. 3). Again, no gadolinium contrast enhancement was observed. Despite immunotherapy, the patient had developed new brain lesions, and we suspected the possibility of PML instead of neurosarcoidosis. Repeated CSF examinations indicated the presence of JCV-DNA (392 copies/mL) at this time; the detected JCV was a prototype with mutations in the regulatory regions of the viral genome characteristic of PML cases. We performed a brain biopsy to confirm the diagnosis and rule out other diseases. Under general anesthesia, a biopsy was performed on the left frontal lobe using the BrainLAB® navigation system (BrainLAB AG, Feldkirchen, Germany). Biopsy specimen sections from the left frontal lobe were analyzed with hematoxylin–eosin and Klüver-Barrera staining. In addition, immunohistochemistry was performed using anti-CD-68, anti-JCV capsid protein VP2/3, and anti-neurofilament/NF-L antibodies. Brain biopsy specimen from the left frontal lobe lesion showed foamy macrophage infiltration and atypical astrocytes by hematoxylin–eosin staining (Fig. 4A, C-D). Klüver-Barrera staining revealed a marked loss of myelin with myelin-laden macrophages in the cerebral white matter (Fig. 4B, E). Axonal staining with an anti-neurofilament/NF-L antibody revealed preserved axonal structure, showing demyelination as the primary pathology (Fig. 4F). Immunohistochemistry demonstrated that the infiltrating cells were CD68^+^ phagocytic macrophages (Fig. 4G). Immunohistochemistry using an anti-JCV capsid protein VP2/3 antibody showed the presence of JCV antibody-positive cells (Fig. 4H). PCR analysis of DNA extracted from the biopsied brain tissue detected the JCV genome (1.03E + 04 copies/cell), confirming the definite pathological diagnosis of PML with no evidence of sarcoidosis-associated granular lesions or HTLV-1-related lymphoma. The possibility of HTLV-1-associated white matter lesions was also considered. However, the pathological findings of the patient was different from previously reported HTLV-1-associated neurological lesions, as lymphocytic infiltrates were reported to be present as well as macrophages [4]. As treatment for PML, prednisolone was reduced to 2.5 mg/day, and mirtazapine was increased to 30 mg/day based on previous reports [5, 6]. We also added oral mefloquine therapy (275 mg/day × 3 days, followed by 275 mg/week) based on previous reports [7, 8], and the patient is currently in a stable state with no disease progression, but with the persistence of aphasia and right hemiparesis.

## Discussion and conclusions

Here, we report an HTLV-1 carrier with untreated sarcoidosis who developed PML after treatment for sarcoidosis. PML is a fatal central nervous system disease caused by the opportunistic infection of JCV due to the suppression of cellular immunity, which leads to subacute neurological symptoms [1]. PML generally occurs in immunocompetent hosts; especially, impairment of cellular immunity leads to JCV reactivation. JCV reactivation is most common in patients with CD4^+^ T cell deficiency, such as HIV infection or idiopathic CD4^+^ cell deficiency syndrome [9]. In addition, CD4^+^ T cell counts reportedly correlate with the prognosis of PML [10]. Conversely, early CD8^+^ T cell reactivity is associated with the prevention and resolution of PML [11].

In this case, HTLV-1 infection was also thought to have contributed to the development of PML. HTLV-1 mainly infects CD4^+^ T lymphocytes and reduces host immunocompetence, even in the non-leukemic state [12]. In addition, lymphocytes infected with HTLV-1 express the *tax* gene. Previous reports showed that JCV promotes intercellular proliferation via the *tax* gene [13], leading to the aggregation and propagation of JCV in CD4^+^ lymphocytes. These mechanisms may increase the risk of developing PML. Previous reports have shown that there is no relationship between HTLV-1 infection and sarcoidosis [14, 15]. In our case, HTLV-1 infection and sarcoidosis independently affected PML. We speculate that sarcoidosis and HTLV-1 infection caused T lymphocyte dysfunction; however, since HTLV-1 proliferates in T lymphocytes, the development of PML may have been suppressed by the maintenance of T lymphocyte counts. Immunotherapy such as steroids and infliximab for sarcoidosis may have decreased T lymphocyte cell count and function; further dysfunction of T lymphocytes may have led to the development of PML.

Concerning the relationship between sarcoidosis and PML, sarcoidosis is reported to be a risk factor for the development of PML, whether treated or not [16]. Clinicians should note that PML with a background of sarcoidosis can easily be misdiagnosed as neurosarcoidosis, and sarcoidosis-associated PML reportedly takes an average of 4.5 months to be diagnosed [17]. In our case, we initially thought the patient had neurosarcoidosis, and it took us 1 month to diagnose PML. To differentiate PML from neurosarcoidosis, CSF analysis is generally normal in PML, while approximately 80% of patients with neurosarcoidosis show abnormal CSF findings [17]. Brain MRI in PML typically shows multifocal asymmetric subcortical white matter lesions without gadolinium enhancement and with T2-high/T1-low signals [18]. In particular, the "milky way appearance" or "punctate pattern" seen on T2-weighted images and post-gadolinium-enhanced T1-weighted images are well-known findings of PML [19, 20]. In the present case, relatively compatible MRI findings were present (Fig. 3C, E), which is, next to the pathological findings, helpful in diagnosing PML. Conversely, neurosarcoidosis is associated with gadolinium-enhanced lesions of the meninges and brain parenchyma [21]. In our case, the possibility of PML was also considered from the onset. We started mirtazapine because initial MRI showed no contrast enhancement, despite CSF examination revealing that JCV-DNA was under the detection limit. We could not find any previous case reports of the coexistence of these three diseases. Here, we report this rare case for the further accumulation of similar cases.

Here, we report a case of PML associated with untreated sarcoidosis and HTLV-1 infection. JCV-DNA was not detected in the initial CSF examination, but was detected after immunotherapy for sarcoidosis. Sarcoidosis and HTLV-1 infection both influence T cells, especially CD4^+^ T cells, and may have been involved in the development of PML. The comorbidity of sarcoidosis, PML, and HTLV-1 infection has not been reported, and is considered a rare event.

## Data Availability

The datasets in this manuscript are available from the corresponding author upon reasonable request.

## Abbreviations

CSF: Cerebrospinal fluid
HIV: Human immunodeficiency virus
HTLV-1: Human T lymphotropic virus-1
JCV: JC virus
MRI: Magnetic resonance imaging
PET: Positron emission tomography
PML: Progressive multifocal leukoencephalopathy
